# A Naturally Occurring Polymorphism in the HIV-1 Tat Basic Domain Inhibits Uptake by Bystander Cells and Leads to Reduced Neuroinflammation

**DOI:** 10.1038/s41598-019-39531-5

**Published:** 2019-03-01

**Authors:** Arthur P. Ruiz, David O. Ajasin, Santhamani Ramasamy, Vera DesMarais, Eliseo A. Eugenin, Vinayaka R. Prasad

**Affiliations:** 1 Department of Microbiology and Immunology, Albert Einstein College of Medicine, Bronx, NY USA; 2 Analytical Imaging Facility, Albert Einstein College of Medicine, Bronx, NY USA; 3 Department of Neuroscience, Cell Biology and Anatomy, University of Texas Medical Branch, Galveston, TX USA; 4 Present Address: Medical Director in Scientific Services, Area 23, New York, NY USA

## Abstract

HIV-1 Tat protein contributes to HIV-neuropathogenesis in several ways including its ability to be taken up by uninfected bystander CNS cells and to activate inflammatory host genes causing synaptic injury. Here, we report that in the globally dominant HIV-1 clade C, Tat displays a naturally occurring polymorphism, R57S, in its basic domain, which mediates cellular uptake. We examined the effect of this polymorphism on Tat uptake and its consequences for cellular gene transactivation. In decapeptides corresponding to the basic domain, a R57S substitution caused up to a 70% reduction in uptake. We also used a transcellular Tat transactivation assay, where we expressed Tat proteins of HIV-1 clade B (Tat-B) or C (Tat-C) or their position 57 variants in HeLa cells. We quantified the secreted Tat proteins and measured their uptake by TZM-bl cells, which provide readout via an HIV-1 Tat-responsive *luciferase* gene. Transactivation by Tat-B was significantly reduced by R57S substitution, while that of Tat-C was enhanced by the reciprocal S57R substitution. Finally, we exposed microglia to Tat variants and found that R57 is required for maximal neuroinflammation. The R57S substitution dampened this response. Thus, genetic variations can modulate the ability of HIV-1 Tat to systemically disseminate neuroinflammation.

## Introduction

HIV-1 infection can result in a spectrum of cognitive and behavioral diseases, termed HIV associated neurocognitive disorders (HAND)^1^. HIV-infected cells in the central nervous system (CNS) release neurotoxic viral proteins (e.g., gp120 and Tat) and a variety of host factors such as inflammatory cytokines, chemokines and small molecules^2,3^. The incidence of HIV associated dementia (HAD), the severe form of HAND, was originally estimated at 15–30% in combination antiretroviral therapy (cART)-naive HIV patients in the US^4^. Widespread cART usage has led to a decreased HAD prevalence to 5–10%^5,6^. There is also a corresponding increase in the prevalence of milder forms of HAND. Overall, HAND is currently estimated at 50% of all HIV-infected individuals^7^. The severity of HAND in the cART era is more closely associated with levels of inflammatory markers and cytokines in the CNS rather than with viremia^7,8^. Therefore, the focus of new HAND therapies is increasingly on the low-level chronic CNS inflammation in HAND patients. This inflammation is due to both infected cell populations and uninfected bystander cells, which can be stimulated by viral proteins such as gp120 and Tat released by infected cells.

HIV Tat protein can be detected in the CNS of patients receiving cART, even with well-controlled peripheral and CNS viral loads^9^. Tat protein plays an important role in neuropathogenesis by recruiting peripheral mononuclear phagocytes (MPs) to the CNS^10,11^, leading to an increased CNS HIV burden. Tat can cause direct neurotoxicity^12^, synaptic loss^13^ and induce host proinflammatory genes^14^. Tat protein is secreted from infected cells by a non-canonical process^15^ and the secreted Tat can be taken up by uninfected bystander cells^16^. Tat uptake is largely mediated by its basic domain^17^. Tat is capable of transcellular signaling^18,19^ in cells relevant to HAND: microglia, macrophages and neurons^20–23^, thereby propagating inflammation beyond the relatively small population of HIV-infected cells in the CNS^24^. Similar to the infected cells, uninfected bystander cells that have internalized Tat can upregulate proinflammatory chemokines and cytokines such as CCL2, TNF-α, IL-2, IL-6, IL-8, IL-1β, and CXCL1 among others^25–31^.

We and others have shown that a naturally occurring polymorphism in Tat, a cysteine to serine substitution at residue 31 (C31S) significantly reduces its neuropathogenic potential, diminishing Tat’s ability to recruit MPs^32^, its neurotoxicity^33,34^ and its pro-inflammatory function^35,36^. We now describe the effects of another natural Tat polymorphism. Tat contains a 10-amino acid basic region from residues 48 to 57, termed the cell-penetrating peptide (CPP) sequence, which mediates Tat uptake by cells. This decapeptide sequence, when covalently linked to a variety of molecular cargoes, facilitates their efficient delivery into cells^37–39^. Tat internalization is mediated by its binding to heparan sulfate proteoglycans (HSPG) ubiquitously expressed on the cell surface. Negatively charged HSPGs coordinate with positively charged arginine and lysine residues in the CPP^40–42^. Substitution of even a single basic residue with an alanine drastically reduces the peptide’s uptake by cells^37^. We previously reported that the R57 Tat residue from non-clade C HIV-1 isolates is well-conserved (67%), while in clade C HIV-1 (HIV-1C), the predominant residue is S57 (86%)^43^. This R57S substitution reduces the number of CPP basic residues (arginine or lysine) from eight in non-clade C Tat proteins to seven in Tat-C. Biological consequences of this substitution are currently unknown. Given intracellular Tat’s ability to modulate transcriptional processes, any polymorphism that can influence its uptake by proximal uninfected cells could have important consequences for systemic production of proinflammatory factors.

In this communication, we have examined the consequences of the naturally occurring R57S substitution on Tat uptake by bystander cells via a wider dissemination of inflammation through activation of proinflammatory genes. First, using fluorescently labeled CPP decapeptides containing either R57 or S57, we demonstrated that CPP-R57 peptide is internalized more efficiently than CPP-S57 (3–4 fold), in a process dependent on CPP binding to extracellular HSPG and endocytosis. We explored the effect of R57S substitution on the uptake of full-length Tat proteins, using a transcellular transactivation assay^18^ where we show a significant reduction. Next, to assess the biological impact of Tat-driven transactivation in the context of HIV neuropathogenesis, we exposed microglial cells to media from Tat-transfected cells, and observed a higher level of induction of proinflammatory cytokine genes – TNFα, IL-6, IL-8, IL-1β and CXCL1 - in response to the more efficient uptake of Tat-R57 when compared to Tat-S57. Finally, spent media from microglia exposed to R57 Tat variants caused a significantly greater neuroinflammation than S57 Tat variants. We conclude that the presence of Tat residue R57 is important in the dissemination of inflammatory response to uninfected bystander cells in the CNS.

## Results

### Polymorphism in the Tat Basic Domain

**Figure 1:**
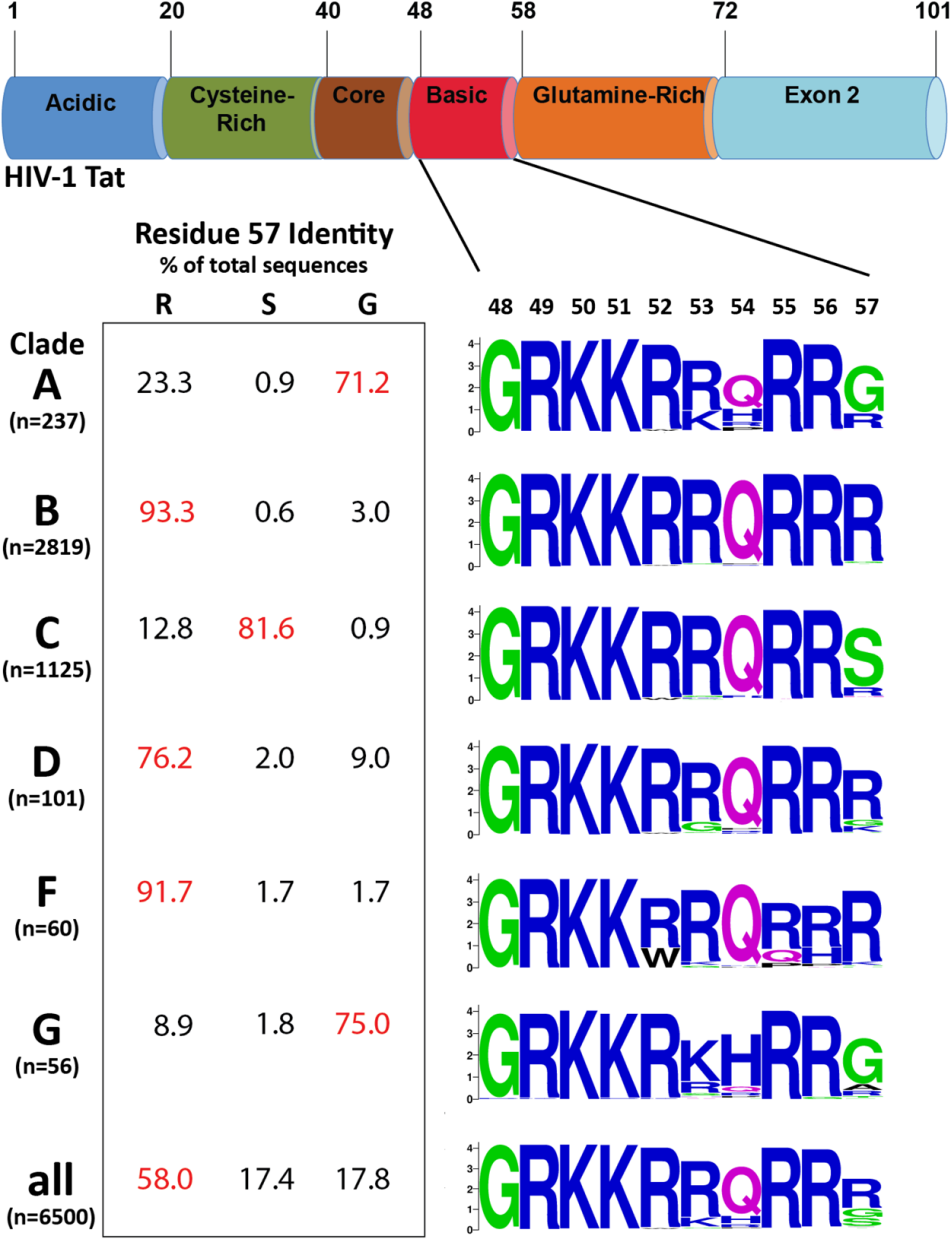
Group M HIV-1 clades display variation in position 57 of Tat CPP sequence. The top panel shows the organization of functional domains in Tat. Tat is a 101 amino acid protein organized into six distinct domains – acidic, cysteine-rich, core, basic, glutamine-rich and exon 2. Numbering at the top is based on HIV-1B Tat. On the lower right are Weblogo consensus sequences of the basic domain or CPP for each clade, with the relative frequency of each residue proportionally represented by font size in the CPP sequence. On the lower left are the frequencies of amino acid residues (R, S or G) at position 57 in Tat protein by clade, with residue frequencies of Tat sequences across clades at the bottom.

Previous work on clade B HIV-1 (HIV-1B) Tat CPP uptake has shown that the 6 arginine and 2 lysine residues in the CPP sequence (R49, K50, K51, R52, R53, R55, R56, and R57) are critical for efficient Tat uptake. The substitution of even a single arginine/lysine with alanine significantly attenuates CPP uptake^37^. We analyzed all HIV-1 Tat exon 1 sequences available in the Los Alamos database. Analysis (Fig. 1) of 6500 CPP sequences across multiple clades shows that among the 8 basic residues in the CPP sequence, the R57 residue was the least conserved. However, when specific clades were examined, R57 was well conserved only in clades B, D and F (93.3%, 76.2% and 91.7% respectively), whereas in clades A, C and G, R57 was respectively present in only 23%, 13% and 9% of the sequences analyzed. Instead, the dominant residue in these clades was either a glycine (clades A and G) or a serine (clade C). Thus, a significant degree of interclade polymorphism is present at HIV-1 Tat CPP residue 57.

### Effect of R57S substitution on the cellular uptake of Tat CPP

**Figure 2:**
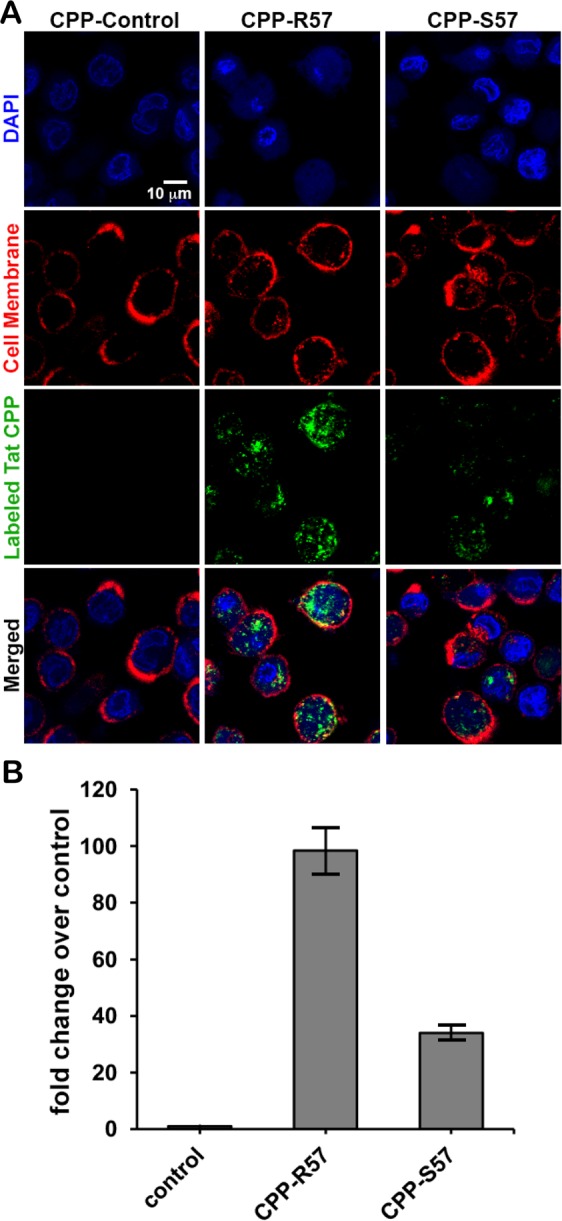
Confocal microscopy reveals differential cellular uptake of Tat-CPP-R57 and Tat-CPP-S57. Incubation of fluorescently labeled Tat-CPP peptides with PMA-differentiated THP-1 cells indicates a greater internalization of CPP-R57 over CPP-S57. (**A**) Confocal fluorescence microscopy images of cells stained for cell nuclei (DAPI, purple), cell membrane (WGA-633, red) or of cells exposed to 1 μM of indicated peptide CPP-control, CPP-R57 or CPP-S57 (green) are shown. Images were captured at 63X magnification. (**B**) The total intensity corresponding to the peptide (green signal) was quantified and presented as a sum of all the cells in 3 fields each for control, CPP-R57 and CPP-S57 and plotted. The control peptide uptake was set at 1 and the corresponding increases for the two experimental peptides as compared to control were plotted.

HIV-1C is the dominant clade in the world, responsible for half of the global HIV burden^44^, and a number of studies have identified genetic features in HIV-1C that attenuate viral functions related to neurovirulence^32–34,45^. Therefore, we chose to investigate the effect of the HIV-1C-specific Tat R57S polymorphism on CPP uptake. We employed two decapeptides corresponding to residues 48–57 in either Tat-B (R57) or Tat-C (S57). Each decapeptide was N-terminally labeled with a fluorescent tag. Differentiated THP-1 monocytic cells were exposed to either CPP-R57 or CPP-S57 at 37 °C for 30 minutes, treated with trypsin to strip away any surface-bound CPP, and imaged by confocal microscopy. Cells exposed to CPP-R57 internalized a higher amount of labeled peptide than cells exposed to CPP-S57 (Fig. 2A,B). A labeled control peptide DAEFRHDSGY displayed background levels of uptake.

**Figure 3:**
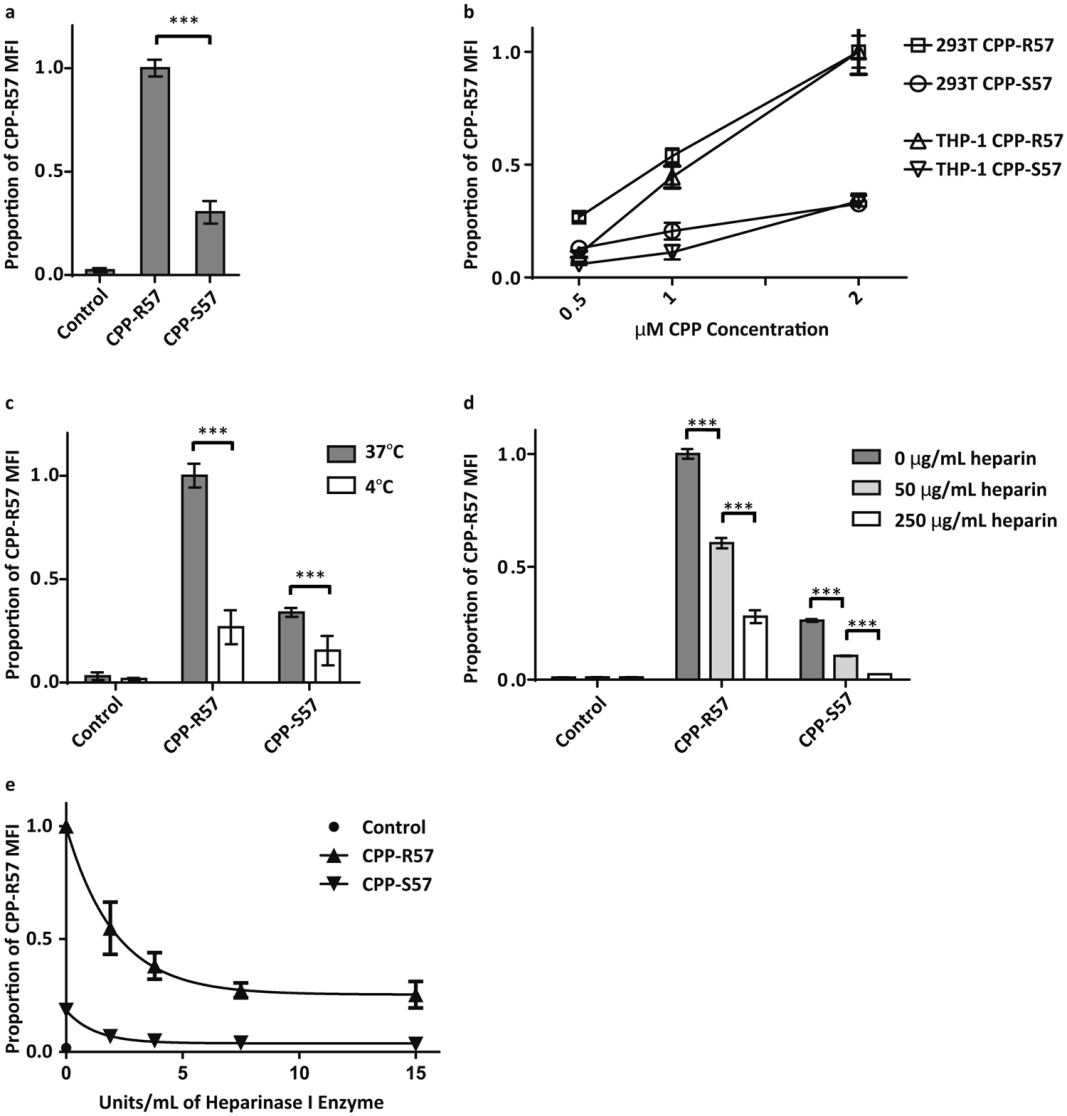
Flow cytometry studies indicate a higher uptake of CPP-R57 over CPP-S57 under a variety of conditions. Various cell types were incubated with fluorescently labeled CPP peptides and the uptake measured by flow cytometry. (**a**) 293T cells exposed to 2 μM of a control peptide, CPP-R57 or CPP-S57 peptides followed by analysis by flow cytometry. Proportional MFI values between experiments (n = 4) were then averaged and plotted on a histogram. Raw MFI values for each sample across different experiments spanned the following ranges: control (1.8–5.5), CPP-R57 (41.9–301.0) and CPP-S57 (11.6–99.0). (**b**) 293T cells and THP-1 cells exposed to increasing concentrations of CPPs as indicated to study dose response. (**c**) 293T cells exposed to 2 μM of CPPs at either 37 °C or 4 °C. Data was combined between different experiments (n = 4), and raw MFI values spanned the following ranges: control (2.8–5.3), CPP-R57 (58.8–266.0) and CPP-S57 (20.0–97.3). (**d**) 293T cells were exposed to CPPs at the indicated concentrations of heparin. (**e**) 293T cells treated for 2 h with the indicated concentrations of Heparinase I enzyme (with Heparinase III enzyme included at 1/17 the concentration of Heparinase I), then exposed to 2 μM of CPP. Data was combined between different experiments (n = 2), and raw MFI values spanned the following ranges: control 0 U (2.1–2.3), CPP-R57 0 U (114.0–145.0), CPP-S57 0 U (18.3–31.9), CPP-R57 1.9 U (64.6–75.1), CPP-S57 1.9 U (7.4–10.9), CPP-R57 3.8 U (46.3–49.9), CPP-S57 3.8 U (6.1–6.8), CPP-R57 7.5 U (34.1–35.4), CPP-S57 7.5 U (5.0–5.2), CPP-R57 15 U (27.8–35.2), and CPP-S57 15 U (4.0–5.0) (***p < 0.001).

We quantified the relative uptake of the CPP variants into 293T cells by flow cytometry and observed that CPP-R57 uptake was significantly higher (~3–4 fold) than CPP-S57 (Fig. 3a). We incubated 293T and the THP1 cells with increasing concentrations of CPP-R57 and CPP-S57 and observed a linear dose response in CPP uptake (Fig. 3b). A reduced uptake of CPP-S57 was seen at all concentrations in both cell lines.

As the cellular uptake of CPPs is mainly achieved by endocytosis^46^, we investigated whether the perturbation of endocytosis would differentially affect the uptake of CPP variants. As endocytosis is an ATP-dependent process that is inhibited at 4 °C, CPP uptake was measured in 293T cells at 4 °C (Fig. 3c) and compared with that at 37 °C. Although CPP-R57 uptake was affected more (~75% decrease) than that of CPP-S57 (50%), treatment at 4 °C did not eliminate the uptake of either peptide, suggesting that a fraction of each peptide may be taken up by a non-endocytic mode^47,48^.

Since Tat CPP uptake is mainly mediated by an interaction with HSPGs at the cell surface^49^, we sought to test the effect of perturbing this interaction on CPP uptake. We first tested the effect of competitive inhibition of CPP uptake by heparin. The 293T cells were incubated with labeled CPPs in the presence of increasing concentrations of soluble heparin. A dose-dependent inhibition of uptake of both CPP-R57 and CPP-S57 was observed in response to increasing amounts of heparin (Fig. 3d). Finally, prior to incubating with CPP, 293T cells were treated with increasing amounts of a combination of Heparinase I and III to remove surface HSPGs. The uptake of both peptides was inhibited in response to increasing levels of pre-treatment with Heparinase enzymes (Fig. 3e). Interestingly, even treatment with very high amounts of Heparinase enzymes did not completely abrogate uptake of either CPP (data not shown), but instead diminished it to a certain baseline level. These results agree with previously reported findings that some Tat CPP uptake may occur by means other than one involving HSPGs^41,46^.

### Transcellular transactivation

Next, we tested the effect of the R57S polymorphism on the cellular uptake of full-length Tat proteins. Recombinant purified Tat protein is notorious for its problems with solubility, aggregation and potential misfolding^50,51^. As a more viable approach, we employed a transcellular transactivation assay, in which HeLa cells are first transiently transfected with equal inputs of expression plasmids encoding Tat protein (equivalent to HIV-infected ‘producer’ cells) from the well-studied strain HIV-1_ADA_ (HIV-1B) and a patient-derived HIV-1_BL-43_ (HIV-1C)^52^. Tat produced within eukaryotic cells is naturally modified, folded and secreted into the medium, rendering it functionally superior to recombinant purified Tat protein^34,53^. Tat-containing media from producer cells are then applied to Tat-responsive TZM-bl cells, which contain an integrated LTR-driven *luciferase* reporter gene (equivalent to uninfected ‘bystander’ cells). TZM-bl cells produce a reporter signal that is proportional to the amount of Tat taken up by cells.

We took several steps to validate the transcellular transactivation assay. Transcellular transactivation is dependent upon the active secretion of Tat from producer cells and its accumulation in the supernatant prior to its uptake. A previous site-directed mutagenesis study identified two motifs critical for its secretion, W11 and an RKK tripeptide (residues 49–51)^15^. Therefore, alanine substitutions were separately introduced at each of the motifs, Tat-B-W11A and Tat-B-49AAA51, and the resulting mutant Tat proteins were tested in our assay. First, TZM-bl reporter cells were directly transfected with these Tat expression constructs, to evaluate any possible intrinsic differences in transactivation function. Tat-B-WT and Tat-B-W11A yielded similar levels of luciferase output upon direct transfection into the reporter cells (Supplementary Fig. S2a) suggesting that W11A substitution does not affect transactivation when it is produced within the cell. However, Tat-B-49AAA51-driven luciferase output was diminished by approximately 90% likely reflecting the essential role of basic residues in TAR-binding and transcriptional transactivation. Therefore, only Tat-B-W11A was useful for testing the impact of perturbing Tat secretion on transcellular transactivation. Exposure of TZM-bl cells to Tat-B-WT produced by HeLa cells transfected with expression constructs led to a robust degree of transcellular transactivation, while Tat-B-W11A led to a ~60% reduction in the level of luciferase induction compared with media containing Tat-B-WT (Supplementary Fig. S2b).

**Figure 4:**
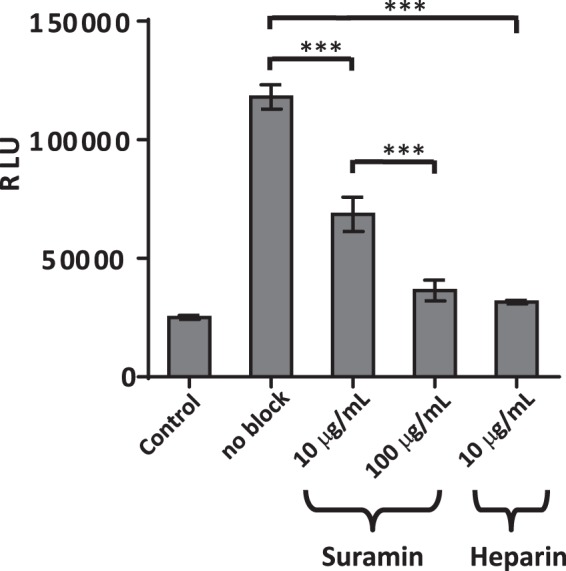
Transcellular transactivation is dependent on the interaction of Tat with HSPGs on the recipient cells. Transcellular transactivation of TZM-bl cells exposed to media collected from HeLa cells transfected with Tat-B, in the presence of indicated amounts of HSPG competitive antagonists Suramin or Heparin.

Since HSPGs mediate Tat uptake, we next investigated whether competitively inhibiting the interaction between Tat and HSPGs can inhibit the subsequent reporter signal. TZM-bl cells were exposed to Tat-B-WT (produced in HeLa cells) in the presence of either heparin or its functional analog suramin^54^. A concentration of 10 μg/ml of heparin was sufficient to diminish transcellular transactivation of extracellular Tat to near background levels, whereas 100 μg/ml of suramin had a similar effect (Fig. 4). Thus, our transcellular transactivation assay is dependent on the HSPG-dependent uptake of secreted Tat, which leads to the luciferase readout.

In order to facilitate the use of equal levels of Tat in the transcellular transactivation, we needed to detect and measure Tat in the supernatants in the transcellular transactivation assay. Therefore, Tat proteins were expressed with a C-terminal c-Myc tag. Both immunoblotting and ELISA were employed. The ELISA involved the use of an anti-Tat monoclonal antibody that recognizes a Tat-C N-terminal epitope in addition to the anti-*myc* antibodies. In order to ensure equivalent detection of all Tat proteins, we introduced mutations in Tat-B expression constructs to create amino acid substitutions R7N and K12N of both the wild type and R57S variants. The mutations did not affect the transcriptional transactivation function of Tat-B (data not shown). The Tat-B variant, optimized for binding to anti-Tat-C antibodies with the R7N and K12N mutations, is termed Tat-B* in our studies.

**Figure 5:**
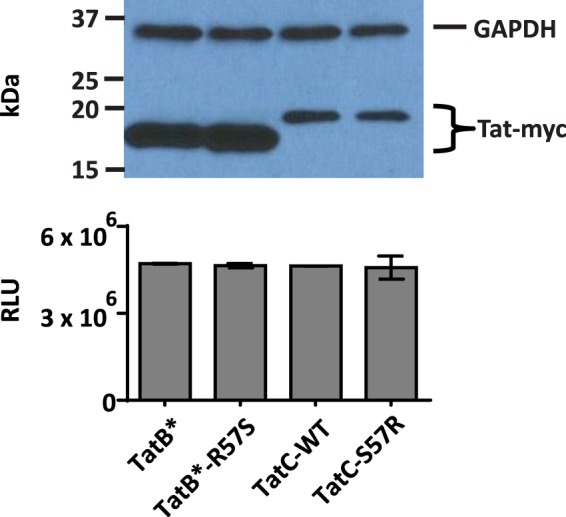
Intracellular levels of Tat protein remain unchanged upon position 57 substitution. Upper panel, Western blot of cell lysates from TZM-bl cells transfected with equal amounts of Tat expression plasmids. Size markers are shown on the left. Lower panel, LTR-Luciferase reporter assays from the lysates of the same cells that correspond to the directly transfected samples in the upper panel (***p < 0.001).

Tat sequences from different clades can vary in their transcriptional transactivation from the LTR. However, residue 57 substitution mutations have been previously shown to affect neither transcriptional transactivation nor nuclear localization^55^. We directly transfected TZM-bl reporter cells with Tat expression constructs with C-terminal *myc* tags – Tat-B* and Tat-B* R57S (HIV-1_ADA_) variant, Tat-C and Tat-C S57R (HIV-1_BL43_) variant. Immunoblot analysis of TZM-bl cells transfected with Tat expression constructs demonstrated that both the Tat-B* variant proteins accumulated to much higher levels than Tat-C and its variant (Fig. 5, upper panel). However, neither the R57S substitution in Tat-B* nor the S57R substitution in Tat-C affected the intracellular levels of the respective Tat proteins when Tat was introduced directly via transfection (Fig. 5, upper panel). LTR-Luciferase reporter assays of TZM-bl cells directly transfected with Tat expression constructs consistently showed that the transfection of equal amounts of Tat expression plasmids results in similar levels of luciferase activity between the Tat variants within a clade (Fig. 5, lower panel). Therefore, despite a lower level of protein detected in cell lysates, Tat-C proteins are able to drive a robust level of Luciferase reporter comparable to a higher level of Tat-B* proteins. This is consistent with previous reports of Tat-C being a more potent transcriptional transactivator than Tat-B^55,56^. Given the differing capabilities of Tat proteins from different clades to drive transcriptional transactivation, we cannot directly use an LTR-reporter signal readout as a proxy for differences in extracellular uptake *between* Tat proteins from different clades. *Within* a clade, however, since residue 57 Tat mutations do not alter either the intracellular accumulation or the transcriptional transactivation of either Tat-B* or Tat-C (Fig. 5), measuring LTR-response from cells exposed to extracellular Tat appears to be a valid way to examine differences in uptake in Tat protein that only differ in residue 57.

**Figure 6:**
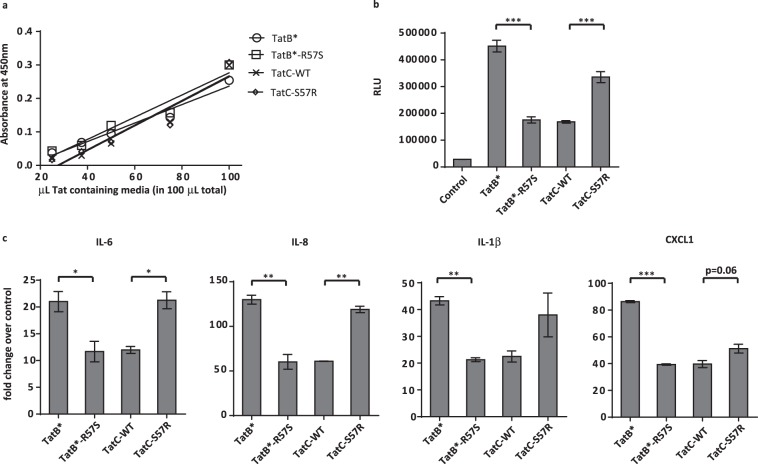
At equivalent inputs, both Tat-B * and Tat-C proteins maximally transactivate only when R57 is present (**a**) Absorbance values from ELISA assay measuring the relative amounts of Tat protein in media samples from HeLa cells transfected with Tat-B*, Tat-B*R57S, Tat-C or Tat-CS57R expression constructs. (**b**) Luciferase activity determined in TZM-bl cells exposed to media from HeLa cells transfected with Tat-B*, Tat-B*R57S, Tat-C or Tat-C S57R expression constructs. The RLUs were normalized using the Tat ELISA absorbance values of transfected HeLa cell media shown in panel (a). Data is representative of 5 independent experiments. (**c**) Induction of cytokine mRNA transcription in C20 microglial cells exposed to media from HeLa cells transfected with Tat-B*, Tat-B* R57S, Tat-C or Tat-C S57R expression constructs. Media samples contained equivalent levels of Tat protein, as determined by the ELISA results in panel (a). Values represent fold-change over expression levels in microglia exposed to untransfected HeLa cell media. Data is representative of 3 independent experiments (*p < 0.05; **p < 0.01; ***p < 0.001).

Due to potential variability in Tat expression of transfected plasmids and the rates of their secretion into the medium, it was necessary to compare levels of extracellular Tat between cells secreting different Tat variants before treating reporter cells. Therefore, we developed a sandwich ELISA, using the C-myc tag and the modified N-termini (to mimic Tat-C). ELISA provided a robust and reproducible means to assess the relative levels of Tat protein variants applied to target cells (Fig. 6a). We were able to use these ELISA absorbance values as correction factors for Luciferase readouts from TZM-bl cells exposed to transfected HeLa cell supernatants. While the R57S substitution in Tat-B* consistently decreased transactivation by a factor of 2 or 3 (~50 to 75%), S57R substitution in Tat-C increased the reporter signal by a factor of 2 (~50%) (Fig. 6b). Given that equal intracellular amounts of the intracellularly expressed wild type and mutant B and C Tat proteins yield equivalent levels of luciferase reporter signal (Fig. 5), the difference in the signal observed between wild type and position 57 variants of Tat (in each clade) observed upon the addition of equal amounts of Tat proteins strongly supports differential uptake.

### Differential Induction of host inflammatory genes by Tat-R57 vs. Tat-S57

As mentioned previously, treatment of various cell types with Tat has been observed to enhance the output of many proinflammatory host factors. We compared changes in proinflammatory cytokine transcript levels in h-Hu_C20 human microglial cells exposed to Tat isogenic variants as a way to assess impact of differential Tat uptake on their inflammatory output. When equivalent levels of Tat proteins were applied to target cells, we observed a pattern of inflammatory gene induction that resembled the transactivation pattern seen in the luciferase reporter gene expression. Tat-B* yielded higher levels of inflammatory cytokine gene transcripts IL-6, IL-8, IL-1β and CXCL1 than Tat-B*-R57S. Conversely, the S57R substitution in Tat-C increased cytokine expression levels (Fig. 6c). The biological impact of increased Tat uptake efficiency, based on the presence of arginine at position 57, therefore, extends to the transcriptional activation of several cytokines implicated in the chronic inflammation that contributes to HAND.

### Effect of Tat uptake on neuroinflammation

**Figure 7:**
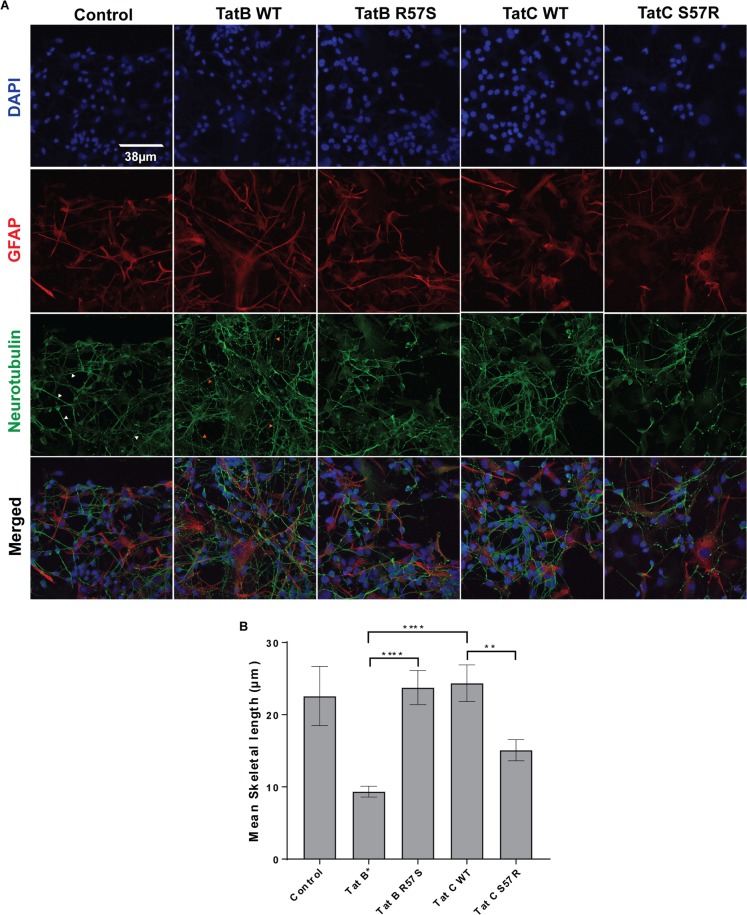
Effect of position 57 polymorphism in Tat B or Tat C (R57S vs. S57R in Tat-B* or Tat C) on neuroinflammation. (**A**) Approximately 50, 000 cells containing a mixed culture of human fetal neurons and glia plated on 35 mm MatTek plates were treated with 200 µl of spent medium from Tat-exposed microglial cultures. The microglia were earlier incubated with spent media from HeLa cells producing Tat-B*, Tat-B*-R57S, Tat-C wild type or Tat-C-S57R. Control panel represents sample derived from expression plasmid lacking *tat*. Neurons were stained with anti-neurotubulin antibodies, astrocytes with anti-GFAP antibodies and nuclei with DAPI. Note that axons/dendrites in Tat-B* samples and Tat-C S57R are shorter and thinner than the other samples and frequently have beaded appearance (red arrowheads in Tat-B* neurotubulin panel), while Control, Tat-B*-R57S and Tat-C wild type samples display thicker and longer axons/dendrites (white arrowheads in control panel) with fewer beaded processes. (**B**) Quantitation of lengths of processes from panel (A) above was done with each of the treatments above and represented as skeletal lengths (using Volocity 6.3). Data is derived from 50 neurons for each treatment (**<0.005; ****<0.0001).

Primary human neuron-astrocyte co-cultures were incubated with spent media from microglia that were exposed to media containing equivalent amounts of Tat variants. The cultures thus treated were fixed and stained for neurotubulin to detect neurons, Glial cell fibrillary acidic protein (GFAP) to detect astrocytes and DAPI to detect nuclei. Processes of neurons (dendrites and axons) exposed to Tat-R57-(Tat-B* and Tat-C S57R)-treated microglial supernatants were shorter and thinner than those treated with Tat S57 (see red arrowheads in TatB WT neurotubulin panels in Fig. 7A). They also displayed a more beaded appearance, indicating neuronal damage, while Tat-B*-R57S and Tat-C-treated neurons displayed thicker and longer dendrites with fewer beaded dendrites (see white arrowheads in Control neurotubulin panels in Fig. 7A).

**Figure 8:**
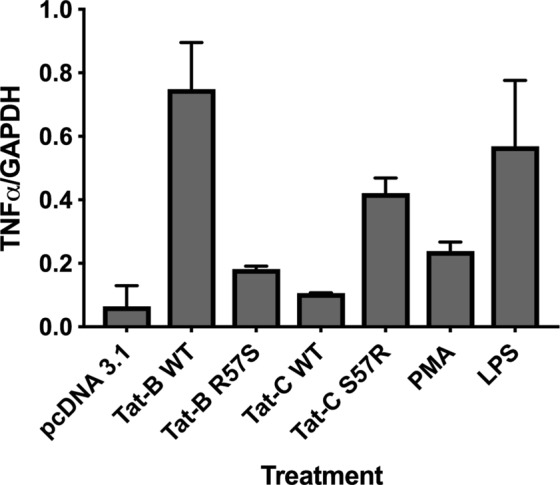
Measuring the TNFα mRNA expression in microglia exposed to Tat variants. C20 microglia were exposed to equivalent amounts of the four Tat variants along with a negative control, 5 ng PMA and LPS positive control media followed by RT-PCR of total RNA and quantification of TNFα PCR products resolved on an agarose gel using densitometry. Band intensities of a housekeeping gene, GAPDH were also estimated in each sample. TNFα band intensities were expressed as a ratio of GAPDH band.

To quantify these effects, we measured the lengths of un-beaded axons/dendrites in the images of 50 neurons in each sample and the control (Volocity 6.3). Beaded axons/dendrites are a representation of neuronal dysfunction and synaptic compromise in several diseases including HAND^57^. We found that the axonal/dendritic integrity is significantly compromised in co-cultures treated with conditioned microglial media with Tat-R57 compared to Tat-S57 variants (Fig. 7B). When we measured TNFα protein levels in the supernatants of Tat-treated microglia (Luminex cytokine detection assay) we detected robust levels of TNFα only in the cases of Tat-B* (19.2 ng/ml) and Tat-C S57R (19.5 ng/ml) samples, compared with their Tat-S57 counterparts – where TNFα was undetectable. Finally, we determined the relative levels of TNFα mRNA via RT-PCR using limited cycles of amplification followed by quantitation of bands in an Agarose gel, which correlates with the TNFα protein levels observed via Luminex measurements (Fig. 8). Thus, there is a direct correlation between Tat uptake, proinflammatory cytokine stimulation and neuroinflammation.

Our results indicate that the presence of an R57 residue allows Tat to be taken up more efficiently by uninfected brain microglial cells, enhancing the production of inflammatory cytokines compared to Tat S57 and propagating neuroinflammation beyond the site of HIV-infected cells.

## Discussion

In this report, we describe the functional effects of a polymorphism at residue 57 in the HIV-1 Tat CPP motif, which has been shown to mediate the uptake of extracellular Tat by bystander/uninfected cells. We used TZM-bl cells, with a Tat-responsive LTR reporter to compare the relative uptake of Tat residue 57 variants with similar transactivation capabilities (i. e., Tat-B*/Tat-B*-R57S; Tat-C/Tat-C-S57R). Potential differences in Tat protein accumulation and/or efficiency of secretion were obviated by measuring the levels of Tat protein variants in the medium via ELISA, which allowed us to normalize the luciferase readout in the TZM-bl to the amount of Tat protein added. When we treated human microglial cells with Tat variants, an R57S substitution reduced the transcriptional transactivation of several proinflammatory cytokine genes. Finally we showed that spent media from microglia exposed to Tat-R57 proteins were more neuroinflammatory compared to microglia exposed to their Tat-S57 counterparts. We propose that the R57S polymorphism has the potential to attenuate CNS neuroinflammation. Furthermore, S57R substitution in a HIV-1C Tat protein reciprocally increased the uptake efficiency of Tat-C protein, transcriptional transactivation and subsequent neuroinflammation.

The importance of the basic domain (CPP) in Tat protein uptake has been well-established^17,49,58^. Tat is known to stimulate proinflammatory gene expression in HIV-infected cells, and it is not surprising that it can activate proinflammatory cytokine genes in bystander cells upon uptake. Thus, any CPP polymorphism that affects Tat uptake can influence the severity of HIV neuropathogenesis by modifying the extent to which the bystander cells take up Tat – and the propagation of a ‘secondary’ inflammatory signal distinct from the infected cells themselves. Our sequence analysis showed that Tat CPP in HIV-1 clades B, D and F largely retain the full complement of eight arginine/lysine residues previously shown to be required for optimal Tat uptake. However, three of the clades, A, C and G displayed either a R57S or a R57G substitution. Confocal microscopy with fluorescently labeled peptides showed that the presence of an arginine residue at position 57 is required for optimal uptake by cells because, both the substitutions - R57S (Fig. 2) and R57G (data not shown) – significantly decreased Tat CPP uptake. In our studies, we focused on R57S and excluded R57G substitution for the following reasons: (i) R57 and S57 residues are present in clade B & D or clade C respectively – which represent important, well-studied clades; (ii) as a proof of principle, we wanted to compare two residues R and S at position 57; (iii) much is known about the neuropathogenesis of clades HIV-1 B (which mainly has Arg at this position) and HIV-1C (which mainly has Ser at this position; (iv) however, literature on the incidence and mechanisms of HIV-1 clade A or clade G –mediated neuropathology is sparse.

Cellular entry of CPP-Tat, with or without R57, takes place largely through endocytosis, since exposure of cells to peptides at 4 °C severely curtailed the uptake of both CPP-R57 and CPP-S57 peptides. However, incubation at 4 °C had a smaller impact on the efficiency of uptake of CPP-S57 (~50% inhibition) than it did on CPP-R57 (~75% inhibition) uptake. It is possible that these results reflect the differential extent to which endocytosis is used as the major mode of uptake for each peptide, depending upon the presence or absence of R57. While endocytosis is the primary route of CPP entry, there is evidence for non-endocytic uptake as well^47,48,59,60^. Additionally, our microscopy results showed that most of the fluorescence signal in the CPP-treated cells was present in discrete vesicular puncta rather than diffusely present in the cytoplasm. This is consistent with previous data indicating the “dead-end” accumulation of Tat-CPP in endosomal vesicles, which lacks other residues in full-length Tat protein required for endosomal escape^61^.

A role of HSPGs in Tat binding to cell surface has been previously established^40–42^. All tissues express HSPGs on the cell surface including brain^62^. Moreover, the human fetal neurons employed here for comparison between the effect of uptake of HIV-1 Tat variants were from same abortus tissue providing a genetically identical target cells in which to compare the damage due to inflammatory signal elaborated by microglia exposed to Tat. Therefore, our experiments are comparing the Tat variants under identical conditions. Thus, secreted Tat protein can be used to measure transcriptional readout from HIV-1 LTR in reporter cells, which can serve as an indicator of Tat uptake efficiency.

Viral signatures associated with the severity of HAND have been previously described. We have reported a naturally occurring C31S polymorphism in Tat dicysteine motif in HIV-1C, which is considered to be important for monocyte chemotaxis function – a property that may underlie the increased monocyte infiltration in CNS that is a hallmark of HAD^43^. *In vitro*^33,35^, virological^63^ and animal model^32^ studies have generated robust evidence that Tat dicysteine motif plays an important role in HIV neuropathogenesis. Tat dicysteine motif facilitates Tat binding to the CCL2 receptor CCR2^45^ to attract more monocytes and macrophages to the CNS sites increasing the inflammatory burden *via* producing a critical mass of HIV-infected macrophages. This dicysteine motif also binds the glutamate receptor NMDA-R^34^ causing increased calcium currents and loss of neuronal termini due to over-firing^64^. While C30S31 mutation does not abrogate transcriptional transactivation from HIV-1 LTR, it disrupts the ability of Tat to bind to CCR2 and NMDA-R and cause neuroinflammation *via* these two distinct mechanisms. Here we describe how a Tat polymorphism can modulate a third pathway of neuroinflammation following the endocytosis of Tat protein leading to transcriptional transactivation of proinflammatory cytokine genes. Our work demonstrates that the induction of inflammatory genes in microglia and subsequent neuronal inflammation from the microglial media is maximized when Tat contains an R57 residue. Thus, the C30C31 motif mediates neurovirulence either directly by binding to NMDA-R or indirectly via binding CCR2 in monocytes or macrophages, while a full complement of basic domain including the R57 residue when present, allows optimal internalization in bystander cells that then leads to transcriptional transactivation of cellular genes. While it is challenging to quantify the relative impact of each of these motifs on neuroinflammation due to the complex modes of action of C30C31 involving different cell types, this would be an important line of future investigation. Neuropathogenic outcomes are likely determined by the net effect of neurovirulence signatures in multiple viral genes in addition to contribution from host factors.

Our findings suggest that Tat R57 is an important viral signature of HAND severity, because of its ability to modulate uptake, which in turn affects expression levels of inflammatory cytokines in bystander cells in the CNS. The data showed that efficient uptake of the R57 Tat leads to a commensurate increase in proinflammatory cytokines being released. It is well-known that proinflammatory cytokines (e.g., TNF-α) are neurotoxic. Thus, our data shows the need for Tat to maintain R57 in the CNS-compartmentalized variants. While we have shown the effect of this polymorphism *in vitro*, further work on the *in vivo* impact of residue 57 polymorphisms on neuroinflammation needs to be conducted employing an *in vivo* model system such as humanized mouse or non-human primate models. Studies comparing *tat* sequences from the CNS of HIV-infected individuals, both with and without HAND, could also reveal whether R57 is associated with HAND. Although such systematic studies have yet to be conducted, we were able to generate some preliminary insight from sequences available at the LANL sequence database. While the overall occurrence of R57 in HIV-1C is 12.8% (Fig. 1; n = 1125), *tat* sequences obtained from the CNS of Indian HIV-1C patients showed a much higher incidence of R57: 70.6%. While the small sample size of this group (n = 17) necessarily limits the strength of any conclusions, we were intrigued by the apparent enrichment of R57 in CNS-derived *tat* sequences from HIV-1C-infected individuals with neurological complications. Comparatively, Tat sequences obtained from brain tissue of HIV-1B patients (n = 69) showed a similarly high incidence of Tat-R57 compared with overall sequences (95.6% and 93.3%, respectively). This information is now presented as a Figure in the Supplementary Data (Supplementary Fig. S3). It is possible that the incidence of HAND in HIV-1C patients is associated with a neurovirulence signature that includes an S57R mutation in Tat. A longitudinal study, following HIV-patients infected with HIV-1B or HIV-1C HIV and correlating compartment-specific signatures of *tat* with neurocognitive deficits could address this question.

Even while cART is able to efficiently suppress CNS viral loads, small amounts of Tat can still be detected in the CNS^9^. This activity of Tat protein can disseminate a constitutive inflammatory signal through bystander cell uptake and activation. Moreover, Tat taken up by neurons can traffic through axonal transport, allowing Tat-mediated inflammation to propagate to sites distal from infected cells^65^. While the full-blown HIV dementia that previously characterized a substantial proportion of uncontrolled infections has largely receded with antiretroviral treatment, the prevalence of milder forms of HAND has significantly increased. The longer lifespans of cART-treated patients mean that their CNS is exposed to a low level of chronic exposure to Tat protein.

Thus, Tat is an attractive candidate for targeted therapy for CNS HIV disease, because of its persistence in virologically suppressed individuals and its ability to elicit inflammation from uninfected cells. The spread of Tat from infected cells to uninfected cells presents a possible target for intervention, whereby agents (such as nucleic acid aptamers or antibodies) that can be delivered into the CNS and that are able to competitively bind Tat could prove effective in diminishing the uptake of Tat by bystander cells. Recent work from S. Valente and coworkers has identified didehydro-Cortistatin A (dCA), a steroidal analog, which is able to efficiently cross the blood-brain barrier, bind to the basic region of Tat and inhibit its ability to transactivate transcription. This group has shown the efficacy of dCA in displacing the nucleolar localization of Tat, and in inhibiting the Tat-mediated dysregulation of inflammatory signaling^66^.

In summary, our results show that the polymorphism at Tat residue 57 can modify the uptake of secreted Tat. The efficiency of Tat uptake by uninfected bystander cells can play an important role in the overall CNS inflammatory burden, which has been shown to be more predictive of HAND severity than viremia levels. Extracellular Tat in the CNS also presents an attractive target for therapeutic intervention designed to diminish chronic inflammatory toxicity and slow or prevent the development of HAND.

## Experimental Procedures

### Antibodies and Enzymes

For Tat detection in ELISA, we used a mouse anti-Tat monoclonal antibody (clone E2.1) raised against a 15 residue N-terminal fragment (amino acid residues 1 to 15) of HIV-1C Tat protein (BL43 strain), a gift from Dr. Udaykumar Ranga (Jawaharalal Nehru Centre for Advanced Scientific Research, Bangalore, India), and a rabbit anti-c-Myc polyclonal antibody (clone A14). The secondary antibody was a goat anti-rabbit HRP-conjugated antibody. For immunoblotting, a mouse anti-c-Myc monoclonal antibody (clone 9E10) and a mouse anti-GAPDH monoclonal antibody (clone GA1R) were used as primary antibodies. The cell surface HSPGs were digested using Heparinase I and Heparinase III enzymes (both cloned from *Bacteroides* strains; NE Biolabs #P0735S and #P0737S respectively).

### Cell lines

The 293T human embryonic kidney cells, HeLa human cervical carcinoma cells, and TZM-bl cells (HeLa cells expressing CD4, CCR5 and an LTR-driven luciferase gene; obtained from the NIH AIDS Reagent Program)^67^ were cultured in DMEM with 10% fetal bovine serum (FBS) and 1% Penicillin-Streptomycin. The h-Hu_C20 microglial cells (Dr. J. Karn, Case Western Reserve University)^68^ were cultured in DMEM/F12 high glucose media (with 5% FBS and 1% Pen-Strep). THP-1 monocytic cell line^69^ was maintained in RPMI media (with 10% FBS, 1% HEPES, and 55 μM β-mercaptoethanol) and differentiated with 10 nM phorbol 12-myristate 13-acetate (PMA).

### Labeled Tat CPP Peptides

Synthetic peptides corresponding to the Tat CPP (amino acid residues 48–57) of HIV-1B (GRKKRRQRRR) or HIV-1C (GRKKRRQRRS), each with an N-terminal fluorescent tag (HiLyte™ Fluor 488) were obtained from AnaSpec Inc. A control peptide of the same length derived from human β-amyloid containing the residues DAEFRHDSGY was selected due to the absence of multiple Arginine and Lysine residues. The control peptide was also tagged with the same fluorescent label. CPPs were diluted to working concentrations in 1% FBS DMEM.

### Confocal microscopy of CPP uptake

Approximately 4 × 10^5^ THP-1 cells were plated in 35 mm MatTek cover dishes in media containing 10 nM PMA to induce adherence and differentiation. After 48 h the media was changed to RPMI/FBS without PMA. Thirty minutes before adding the labeled-CPPs, cell media were replaced with low serum (1% FBS) RPMI. THP-1 cells were treated with CPP for 30 minutes at 37 °C, washed twice with phosphate buffered saline (PBS) in 10% FBS, and incubated with 0.05% trypsin for 10 minutes to digest any surface-bound CPPs. Cells were washed twice with PBS/FBS and then incubated for 20 minutes in Hank’s Buffered Salt Solution (HBSS) with 1X diluted WGA-633 cell membrane stain (Life Technologies). Cells were washed two additional times with PBS/FBS, then fixed in 4% paraformaldehyde (PFA in PBS) for 15 minutes, washed twice with PBS/FBS, and finally immersed in PBS/FBS containing DAPI (Life Technologies) at 1 μg/ml, to stain the cell nuclei. Cells were imaged using a Leica SP8 Confocal Microscope and collected fluorescence data from 405 nm (DAPI stained cell nuclei), 488 nm (internalized labeled-CPP signal), and 633 nm (WGA-633 stained plasma membrane) channels as a series of Z-stacks. Analysis of cells with red and green fluorescence was conducted using Image J. The perimeter of each cell was circled using the red stain as a guide. Subsequently, mean intensity of green fluorescence contained in each cell was measured. For each condition, three random fields of view were analyzed. For control, R57 and S57, a total of 146, 81 and 119 cells were analyzed.

### Flow Cytometry

293T cells were plated in 12-well plates at 2.5 × 10^5^ cells per well, to achieve approximately 70% - 80% confluence by the next day. Thirty minutes before the experiment, cell medium was changed to low-serum DMEM with 1% Pen-Strep. Cells were washed once with PBS, treated with Tat-CPP in low-serum DMEM, and placed in either a tissue-culture incubator (37 °C/5% CO_2_) or a refrigerator (4 °C)(samples treated at 4 °C were pre-incubated in refrigerator for 10 minutes before CPP treatment). Cells were gently washed twice with PBS (w/10%FBS), then treated with 300 µl of pre-warmed 0.05% trypsin for 5 min, to detach cells and enzymatically digest surface-bound Tat-CPP. Cells were resuspended in 1 ml PBS (with 10% FBS and 2 mM EDTA), and peptide uptake assessed by flow cytometry (FACScalibur). Live cells were gated using forward-scatter and side-scatter ratio, then interrogated >10,000 live-cell events each for fluorescence signal emission at 488 nm. Data was analyzed using FlowJo software v9.3.3 (FlowJo LLC). To adjust for differences in instrument settings and voltage adjustments between experiments we transformed the raw MFI values within each experiment into values proportional to the greatest MFI reading in that particular experiment. Heparinase enzymes were used to pre-treat cells by combination of Heparinase I and III at 17 IU and 1 IU, respectively, per mL of plain DMEM. Cells were treated with enzymes for 2 h in an 37 °C, then washed with PBS and exposed to CPPs as above. Successful Heparinase digestion was confirmed by the release of oligosachharide products containing unsaturated uronic acids, which were detected in the supernatants by UV spectroscopic absorption at 232 nm.

### Tat expression constructs

Sequences encoding HIV-1 Tat protein variants employed in this study, Tat-B (HIV-1_ADA_) and Tat-C (HIV-1_BL43_), were cloned into the mammalian expression plasmid *pcDNA3*.*1(* + ) behind a CMV promoter. Site-directed mutagenesis was used to create codon 57 substitutions in each *tat* gene, to express Tat-B-R57S (5′-GGAGACAGCGACGAAGCACTCCTCAAGACAGTC3′) or Tat-C-S57R (5′AGACAGCGACGAAGAGCTCCTCCAAGCAG3′). Two other substitutions, a W11A (5′-TCCTAGACTAGAGCCCGCGAAGCATCCAGGAAGC3′) and a triple alanine substitution in residues 49-RKK-51 (5′AGGCTTAGGCATCTCCTATGGCGCGGCGGCGCGGAGACAGCGACGAAGAACT-3′) were introduced in the HIV-1B *tat* gene. At the 3′ end of *tat* sequences (both HIV-1B and HIV-1C as well as their codon 57 substitutions), a sequence coding for a GGSG tetrapeptide linker (5′GGAGGATCCGGA3′) was appended followed by a sequence coding for a C-terminal *myc* protein tag (5′GAACAAAAACTTATTTCTGAAGAAGATCTG3′) to facilitate the detection of secreted extracellular Tat by ELISA. Furthermore, to endow the Tat-B proteins with the same antibody epitope as Tat-C, to facilitate equal antibody recognition during ELISA, mutations were created in plasmids coding for Tat-B-WT and Tat-B-R57S at amino acid residues 7 (Arg to Asn) and 12 (Lys to Asn) respectively using site directed mutagenesis (5′GCCATGGAGCCAGTAGATCCTAACCTAGAGCCCTGGAATCATCCAGGAAGCCAGCC3′). Tat proteins with these N-terminal mutations are referred to in the text as Tat-B*.

### Trans-cellular transactivation by Tat

HeLa cells were seeded in 6-well plates at 7.5 × 10^5^ cells per well and transfected with 2.5 μg of Tat expression plasmid DNA (Lipofectamine 3000, Invitrogen). After 6 h, the media was replaced with DMEM/10% FBS. Next day, TZM-bl reporter cells were seeded in a 24-well plate, at 1.5 × 10^5^ cells per well. Similarly, C20 microglial cells were seeded in a 12-well plate, at 3 × 10^5^ cells per well. After allowing 5–6 h for cell attachment, supernatant from the *tat*-transfected HeLa cells was collected, which was centrifuged at 500 × *g* for 10 minutes to remove debris and dead cells. Importantly, at this step and all others involving handling of Tat-containing media, siliconized tubes and tips were used to minimize the adsorption of Tat to surfaces. *Tat*-transfected HeLa supernatants were used to replace either TZM-bl media (400 μL) or C20 media (800 μL), and the cells incubated for 12 h (C20) or 24 h (TZM-bl).

### Direct Transfection of Reporter Cells

Approximately 1.3 × 10^5^ TZM-bl reporter cells were seeded in a 24-well plate, in order to achieve ~80% confluency by the next day, and then transfected with varying amounts (between 125 ng to 1000 ng) of Tat expression plasmid DNA. After 6 h, the media were removed and replaced with DMEM media. After 18 h, the TZM-bl cells were harvested as described below.

### Luciferase Assay

TZM-bl cell lysates were harvested and stored at −80 °C until luciferase activity was measured. Frozen TZM-bl cell lysates were thawed on ice for 30 minutes, centrifuged at 14,000 × *g* for 2 minutes, and the protein concentration in the supernatants measured by BCA protein assay^70^. For each sample, 50 µL of lysate was combined with 50 µL of room-temperature reconstituted Luciferase Assay Substrate (Promega) in a well of 96-well white-bottomed plate. The side of the plate was gently tapped and each sample was immediately assayed for luciferase activity using a plate-reader, measuring each sample in Relative Light Units (RLU) (PerkinElmer VICTOR-2).

### Quantitative Real Time PCR of cytokine gene expression in C20 cells exposed to Tat

We transfected 2 × 10^6^ HeLa cells with pEGFP, pcDNA 3.1 and the plasmids expressing the four Tat variants. At 24 hours post transfection, media were collected and Tat ELISA performed to determine the amount of Tat present. C20 microglia (2 × 10^6^) were incubated with equal amounts of Tat media for 24 h. PMA (5 ng) or LPS (5 ng) were also included in separate wells as controls. C20 cells were washed with PBS, treated with trypsin, pelleted by centrifugation at 500 × *g* for 10 minutes, and total cell RNA isolated using an RNeasy Kit (Qiagen). RNA was reverse transcribed in a first-strand cDNA synthesis reaction (GoScript, Promega), using oligo dT primers. The cDNA was diluted to 100 ng per 3 μL in each reaction well, and combined with 2x Taqman Gene Expression Master Mix (ThermoFisher) and Taqman Gene Expression Assay primer-probes: GAPDH (Hs02758991_g1), IL6 (Hs00985639_m1), IL8 (Hs00174103_m1), IL-1β (Hs00174097_m1) or CXCL1 (Hs00236937_m1). Each sample was assayed in triplicate on a 384-well plate, and run under standard qRT-PCR cycling conditions on a 7900HT instrument (ABI).

We also performed PCR to quantify TNFα gene expression in C20 cells exposed to Tat. Briefly, equal inputs of Tat containing media from HeLa cells were added to C20 cells. The cDNAs synthesized from the total RNA extracted from the microglia that were exposed to equal amounts of Tat protein were used to PCR amplify TNFα (forward primer: 5′GAAAGCATGATCCGGGACGTG3′ and reverse primer: 5′GATGGCAGAGAGGAGGTTGAC3′ and GAPDH (forward primer: 5′TGGAAGGACTCATGACCACA3′ and reverse primer: 5′AGGGGTCTACATGGCAACTG3′) cDNA sequences. The products were resolved on a 1.5% agarose gel and the band intensities from three separate experiments were quantified by densitometry using NIH Image J and the mean intensities plotted.

### Sandwich ELISA for Tat

In a 96-well flat-bottom tissue culture plate (Corning) 100 μL of anti-Tat antibody E2.1 was placed at a concentration of 0.002 μg/μL in antibody coating buffer (0.15 g Na_2_CO_3,_ 0.29 g NaHCO_3_ in 100 ml distilled water, pH 9.6). The plate was sealed and gently rocked overnight at 4 °C. On Day 2, the wells were washed 3 times in wash buffer (0.05% Tween in PBS), then blocked for 1 h at room temperature in 100 μL blocking buffer (wash buffer with 2.5% bovine serum albumin). Media from *tat-*transfected HeLa cells was harvested and centrifuged as above, then serially diluted using full DMEM media. The blocking buffer was aspirated from the plate and 100 μL of Tat-containing supernatant was added at different dilutions. A negative control sample of DMEM media was also included. The plate was sealed and gently rocked overnight at 4 °C. On Day 3, the wells were washed in wash buffer 3 times. Anti-Myc antibody A14 was diluted to 0.004 μg/μL in blocking buffer, and 100 μL added to each well. The plate was sealed and rocked gently for 1 h at room temperature. The plate was washed in wash buffer 6 times, then 100 μL of anti-rabbit HRP-conjugated secondary antibody (diluted 2000X in wash buffer) was added to each well. The plate was sealed and rocked gently for 30 minutes at room temperature. The plate was washed in wash buffer 3 times, and then 100 μL of room temperature TMB was added to each well. After 15 minutes incubation at room temperature, 100 μL of 0.2 N H_2_SO_4_ was added to each well, and absorbance at 450 nm measured using a plate reader (PerkinElmer VICTOR-2). Sample absorbance values were adjusted by subtraction of the absorbance value from a negative DMEM control sample.

### Tat CPP sequence alignment

All available Tat exon 1 sequences were retrieved from the HIV Sequence Database at the Los Alamos National Laboratory (LANL; www.hiv.lanl.gov/). Each search was filtered by clade and one sequence (top hit) per unique patient was retained. Sequences were translated to amino acid sequences, and then sequences aligned using Geneious software v6.1.7 (Biomatters Ltd.) to extract residues 48–57 for each sequence. These extracted CPP sequences were uploaded to a Weblogo tool^71^ (http://weblogo.berkeley.edu/logo.cgi) to generate CPP consensus sequence for each clade, with proportional representation of each residue. The sequences were retrieved, sorted and tabulated in 2016.

### Treatment of neurons with spent media from Tat-exposed microglia

C20 microglia were exposed for 24 h, to equivalent inputs of HeLa cell media containing Tat protein (Tat-B*, Tat-B* R57S, Tat-C or Tat-C S57R or empty vector) as described above for cytokine expression. As a control, spent media of HeLa cells transfected with an empty pcDNA 3.1 construct was used. Approximately 50, 000 cells containing a mixture of human fetal neurons and glia plated on 35 mm MatTek plates were treated with 200 µl of spent media from Tat-exposed microglial cultures for 30 h.

For the purpose of imaging of neurons and quantifying axonal/dendritic lengths, neurons treated as above were fixed with 4% PFA for 15 min and washed with 1x PBS. Cells were permeabilized with 0.1% Triton X-100 for 3 minutes and washed as before. Non-specific sites were blocked with 1X SBS buffer (50 mM EDTA, 0.01% Fish Gelatin, 0.1 g BSA, 1% Horse serum in PBS) for 1 h at room temperature and the cells washed. Cells were stained with anti-neurotubulin antibodies, anti-GFAP antibodies and DAPI to stain neurons, glial cells and nuclei respectively. Neuronal images were captured using a Leica Confocal TCS SP5 microscope (Leica Microsystems, Bannockburn, IL). Axonal/dendritic lengths were quantified using Volocity 6.3 software.

## Supplementary information

Supplementary Data File

## Data Availability

The datasets generated during and/or analyzed during the current study are available from the corresponding author on reasonable request.
