# Supplementary material for: A Naturally Occurring Polymorphism in the HIV-1 Tat Basic Domain Inhibits Uptake by Bystander Cells and Leads to Reduced Neuroinflammation
Source: Sci Rep. 2019 Mar 1;9:3308. doi: 10.1038/s41598-019-39531-5 (PMC6397180; doi:10.1038/s41598-019-39531-5)
Supplement: Supplementary file 1 — Supplementary Data File [file 41598_2019_39531_MOESM1_ESM.pdf]

Supplementary Data

Manuscript Title: A Naturally Occurring Polymorphism in the HIV-1 Tat Basic Domain Inhibits Uptake by Bystander Cells and Leads to Reduced Neuroinflammation

Authors: Arthur P. Ruiz, David O. Ajasin, Santhamani Ramasamy, Vera DesMarais, Eliseo A. Eugenin and Vinayaka R. Prasad

Supplementary Figure S1 *Confocal microscopy showing differential cellular uptake of Tat-CPP-R57 and Tat-CPP-S57.* The experiment is the same as that presented in Figure 2. However, a larger field is presented to be able to view the differential uptake in a larger number of cells. Images represent confocal microcscopy images of differentiated THP-1 cells incubated with fluorescently labeled Tat-CPP peptides. Images of cells stained for cell nuclei (Hoechst Blue), cell membrane (WGA-633, red) or cells exposed to 2.5 μM of indicated peptide - CPP-control, CPP-R57 or CPP-S57 (green) are shown. Images were captured at 63X magnification.

Supplementary Figure S2 *Transcellular transactivation is dependent on the secretion of Tat from producer cells.* **(a)** TZM-bl cells were directly transfected with expression plasmids encoding Tat-B, Tat-B W11A or Tat-B 49AAA51 to determine intrinsic differences in LTR transactivation. **(b)** Transcellular transactivation of TZM-bl cells exposed to media collected from HeLa cells transfected with the Tat expression plasmids indicated in panel (a) above. The mutant 49AAA51 was not included in this assay as it displayed only 10% of the TatB-WT transcriptional activity.

Supplementary Figure 3 – Comparison of Tat residue 57 identify between clades in sequences from whole body vs CNS. All available Tat exon 1 sequences from the Los Alamos National Laboratories were filtered in the 'Sample Tissue' option for either 'any' or 'CSF'+'brain'. Nucleotide sequences were retrieved (one unique sample per patient), translated to polypeptide, and residue 57 identities extracted and quantified.

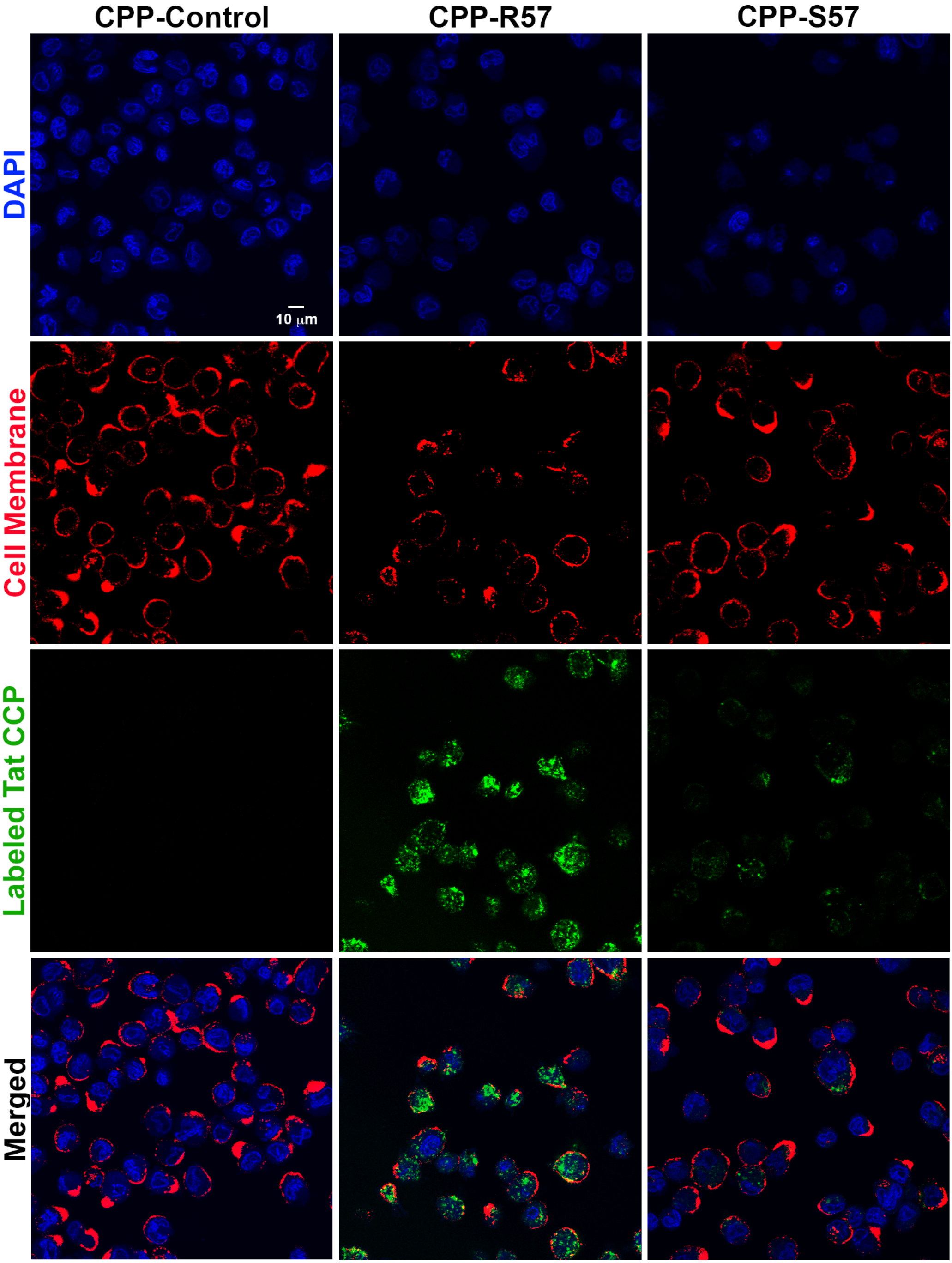

CPP-Control
CPP-R57
CPP-S57
DAPI
Cell Membrane
Labeled Tat CCP
Merged
10 μm

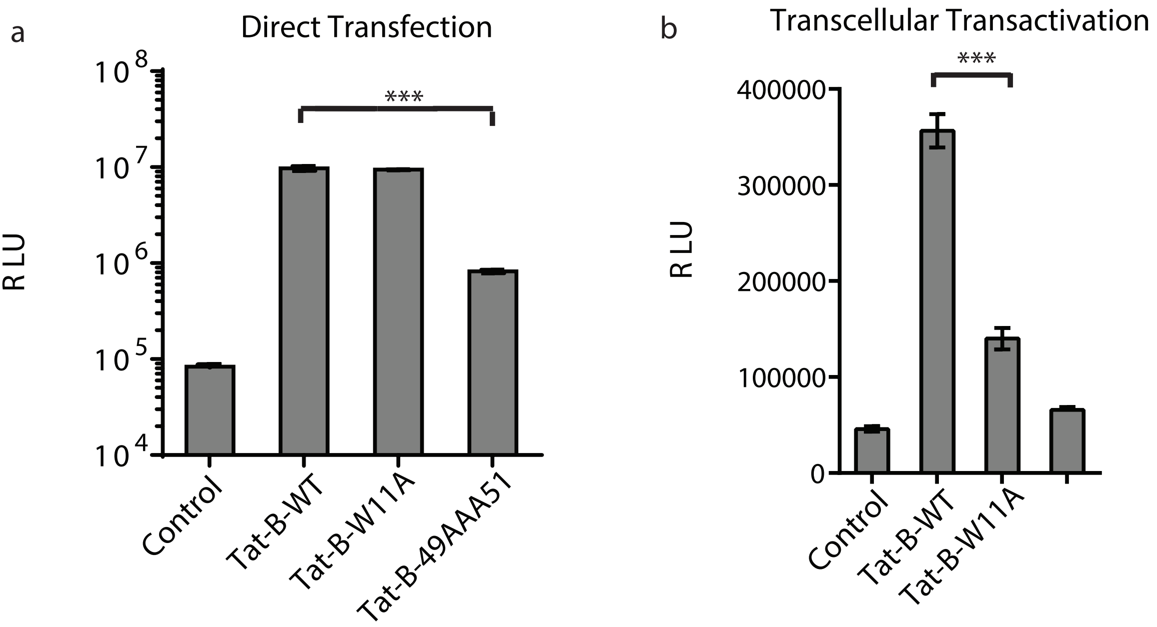
a
Direct Transfection
10^8
10^7
10^6
10^5
10^4
R LU
***
Control
Tat-B-WT
Tat-B-W11A
Tat-B-49AAA51
b
Transcellular Transactivation
400000
300000
200000
100000
0
R LU
***
Control
Tat-B-WT
Tat-B-W11A

Residue 57 Identity

HIV-1 clade:	all sequences R	all sequences S		CNS-derived sequences R	CNS-derived sequences S
B (n=2819)	93.3%	0.6%	B (n=69)	95.6%	0%
C (n=1125)	12.8%	81.6%	C (n=17)	70.6%	23.5%
